# Leptin and Adiponectin as Uremic Adipokines: Associations with Survival in a Prospective Hemodialysis Cohort

**DOI:** 10.3390/toxins17110525

**Published:** 2025-10-25

**Authors:** Thuy-Anh V. Bui, Amy S. You, Sara S. Kalantar, Jihoon Yoon, Yoko Narasaki, John Sy, Ramy Hanna, Andrea Daza, Yalitzi Guerrero, Anyssa Dang, Ria Arora, Danh V. Nguyen, Kamyar Kalantar-Zadeh, Connie M. Rhee

**Affiliations:** 1 Baylor College of Medicine, Houston, TX 77030, USA; 2 Division of Nephrology, Hypertension and Kidney Transplantation, University of California Irvine, Orange, CA 92868, USA; 3 Department of Medicine, David Geffen School of Medicine at UCLA, Los Angeles, CA 90073, USA; 4 University of California Berkeley, Berkeley, CA 94720, USA; 5 Nephrology Section, Veterans Affairs Greater Los Angeles Healthcare System, Los Angeles, CA 90073, USA; 6 Barnard College of Columbia University, New York, NY 10027, USA; 7 Division of General Internal Medicine, University of California Irvine, Orange, CA 92868, USA; 8 Lundquist Institute at Harbor-UCLA, Torrance, CA 90509, USA; 9 Tibor Rubin Long Beach Veterans Affairs Medical Center, Long Beach, CA 90822, USA

**Keywords:** chronic kidney disease, hemodialysis, leptin, adiponectin, adipokines

## Abstract

Background: While experimental models show that leptin and adiponectin have inverse effects on the cardiovascular system, it has been suggested that the leptin-to-adiponectin (L/A) ratio may be an important predictor of cardiovascular disease and death. Higher circulating leptin and adiponectin levels are observed in uremia due to decreased renal degradation and/or clearance and increased production. We sought to examine the association between the L/A ratio and mortality in a prospective hemodialysis cohort. Methods: Among a prospective cohort of 448 hemodialysis patients from the NIH “Malnutrition, Diet, and Racial Disparities in Chronic Kidney Disease (CKD) (MADRAD) study who underwent leptin and adiponectin measurements, we examined characteristics associated with high leptin and adiponectin (defined as the highest tertile) using logistic regression. We then examined the association of L/A ratio levels (categorized as tertiles) with all-cause mortality using Cox regression. Results: Multivariable logistic regression analyses showed female sex, diabetes, presence of an arteriovenous fistula/graft, and lower serum albumin, IL-6, and adiponectin were associated with high leptin, whereas female sex, longer vintage, Black race, higher IL-6, and lower leptin were associated with high adiponectin. When examining L/A ratios, the highest tertile was associated with lower mortality in case-mix Cox models (ref: lowest tertile): HR (95% CI) 0.14 (0.06–0.35). These associations were robust in analyses that additionally adjusted for laboratory covariates: (HR 95% CI) 0.18 (0.07–0.46). Conclusions: In a prospective cohort of hemodialysis patients, inflammation and malnutrition markers were associated with lower leptin and higher adiponectin levels. Additionally, high L/A ratio levels were associated with lower mortality. Further studies are needed to determine the mechanisms relating adipocytokines, inflammation and nutrition, and survival in this population.

## 1. Introduction

Adipose tissue has gained recognition as an active endocrine organ that secretes cytokines called adipocytokines (also known as adipokines) to regulate metabolic status. Two of the most prominent hormonally active adipokines are leptin and adiponectin [1]. Leptin is a 167-amino acid, 16 kDa protein product of the obese (ob) gene that binds to receptors in the hypothalamus, where its signals lead to the suppression of appetite to regulate metabolism. Leptin also regulates other neuroendocrine factors and is associated with promoting inflammation [2] before its metabolic degradation in the renal tubules [3]. Adiponectin is a 240-amino acid adipokine that is synthesized as a 28–30 kDa monomer and is assembled into low molecular weight (LMW) and high molecular weight (HMW) forms [1,4]. In contrast with leptin, it has anti-inflammatory as well as insulin-sensitizing properties. Ultimately, it is metabolized by the liver and eliminated by the kidneys [5].

In the general population, leptin levels directly correlate with body mass index [6] and have been associated with adverse cardiovascular outcomes. Increased body fat composition and high leptin occurrence are attributed to leptin resistance in obesity [7,8,9]. Leptin is defined as pro-atherogenic, which corresponds with its association with a higher risk of cerebral vascular disease, carotid intimal hyperplasia, and cardiovascular disease [10,11,12,13]. Conversely, adiponectin levels are low in populations with high body fat [14], type 2 diabetes [14], and coronary heart disease [15], suggesting its anti-atherogenic impact. Serum concentrations of adiponectin are lower in obesity, whereas adiponectin levels significantly increase with weight loss as well as with insulin sensitivity.

In the chronic kidney disease (CKD) population, including those receiving dialysis, higher levels of leptin have been observed, presumably due to decreased renal degradation and clearance, increased production, and uremic factors [16]. In contrast to the general population, limited data suggest that low leptin levels are associated with higher death risk in CKD patients [17,18], while there are conflicting reports of the relationships between elevated leptin and cardiovascular outcomes as the leading cause of death in this population. Some studies have observed associations between serum leptin levels and death from cardiovascular events [19,20], while others did not observe this link [21,22]. Additionally, higher adiponectin levels have also been observed in uremia, likely due to impaired renal clearance and as a compensatory response to metabolic disturbances and inflammation [23]. Higher circulating levels of adiponectin have been associated with higher all-cause mortality risk in both hemodialysis patients [24] and kidney transplant recipients [25]. However, the relationship between adiponectin and survival in CKD remains unclear owing to mixed observations [26].

Notably, emerging data suggest that the ratio of leptin-to-adiponectin (L/A) levels may be a better indicator of the risk of cardiovascular disease and mortality in CKD [27,28], as it holistically accounts for both pro-atherogenic leptin and anti-atherogenic adiponectin concentrations. To date, there has only been one study in the CKD population examining L/A ratios and patient survival [29], but inference from this study is limited by its small cohort size, focus on peritoneal dialysis patients only, and lack of representation from a diverse racial and ethnic population. To better elucidate the impact of the uremic adipokines, leptin and adiponectin, in the end-stage kidney disease population, we sought to examine the associations of serum L/A ratios and mortality risk in a well-characterized, diverse, multi-center prospective cohort of hemodialysis patients from the NIH Malnutrition, Diet, and Racial Disparities in Chronic Kidney Disease (MADRAD) (NCT01415570) study.

## 2. Results

### 2.1. Cohort Description

A total of 448 hemodialysis patients met the eligibility criteria for this study (Supplemental Figure S1), and the mean ± SD age of this cohort was 55 ± 14 years. Among this cohort, 31% were of Black and 50% were of Hispanic racial/ethnic background, and 55% had underlying diabetes. L/A ratios were calculated for the cohort, and the mean ± SD, median, and minimum–maximum range of serum L/A ratios in the cohort were 6.0 ± 12.4, 1.1 (0.3, 5.7), and 0.003–102.0 µg/mL, respectively. The mean ± SD, median, and minimum–maximum range of serum leptin concentrations in the cohort were 50.3 ± 75.8, 16.5 (5.7, 54.6), and 0.07–401.5 µg/mL, respectively. The mean ± SD, median, and minimum–maximum range of serum adiponectin in the cohort were 17.7 ± 11.3, 15.2 (9.1, 24.2), and 1.6–79.5 µg/mL, respectively. A scatterplot of leptin vs. adiponectin levels and overlying regression line (adiponectin = 19.90385 − 0.0443667 × leptin) is shown in Supplemental Figure S2.

Compared with patients in the lowest L/A ratio tertile, patients in the highest L/A ratio tertile were more likely to be female, were less likely to be of Black race or Hispanic ethnicity, tended to be of younger dialysis vintage, and were more likely to have diabetes (Table 1). Baseline characteristics of patients categorized by leptin and adiponectin tertiles are shown in Supplemental Tables S1 and S2.

### 2.2. Clinical Characteristics Associated with Adipokine Levels

We examined clinical characteristics associated with higher leptin and adiponectin levels (separately). In “case-mix + laboratory”-adjusted logistic regression analyses, we observed that female sex, diabetes, presence of an arteriovenous fistula/graft, and lower serum albumin, IL-6, and adiponectin levels were significantly associated with the highest tertile of leptin (Table 2). In logistic regression analyses adjusted for case-mix and laboratory covariates, we found that female sex, longer dialysis vintage, Black race, higher IL-6 levels, and lower leptin levels were significantly associated with the highest tertile of adiponectin (Table 3).

In Spearman correlation analyses adjusted for case-mix + laboratory covariates, adiponectin and IL-6 had significant inverse correlations with leptin levels (Supplemental Table S3). In contrast, Spearman correlation showed that longer dialysis vintage and higher IL-6 levels had significant positive correlations with adiponectin levels, whereas leptin had negative inverse correlations with adiponectin levels (Supplemental Table S4).

### 2.3. Leptin-to-Adiponectin Ratio and Mortality Risk

**Figure 1:**
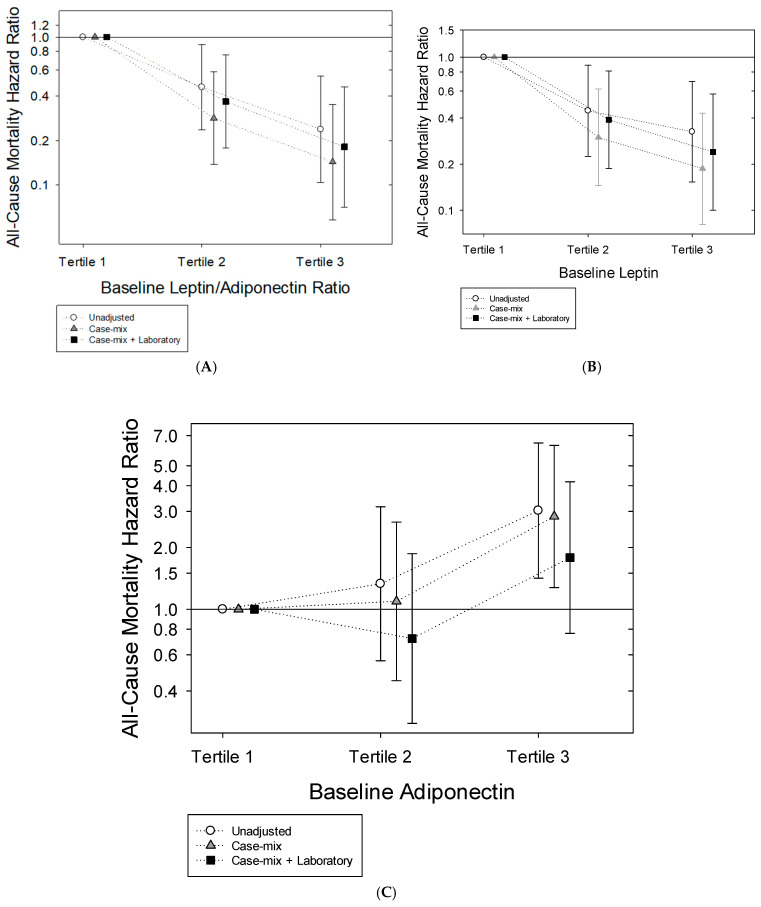
Associations between leptin-to-adiponectin ratio (Panel (**A**)), leptin (Panel (**B**)), and adiponectin (Panel (**C**)), categorized as tertiles, with all-cause mortality risk (hazard ratio with 95% confidence interval) in hemodialysis patients.

In unadjusted Cox regression analyses, we observed that incrementally higher tertiles of L/A ratios were associated with increasingly greater survival in comparison to the lowest tertile of L/A ratios: HR (95% confidence interval (CI)) 0.46 (0.24–0.89) and 0.24 (0.10–0.55) for the middle and highest tertiles, respectively (Figure 1A and Supplemental Table S5). Following adjustment for case-mix covariates, we found that the magnitude of survival benefit was amplified: adjusted HRs (aHRs) (95% CIs) 0.28 (0.14–0.58) and 0.14 (0.06–0.35) for the middle and highest tertiles, respectively. Further adjustment for case-mix + laboratory covariates showed a robust pattern of associations: aHRs (95% CIs) 0.37 (0.18, 0.76) and 0.18 (0.07, 0.46) for the middle and highest tertiles, respectively. Sensitivity analyses that incrementally adjusted for expanded case-mix + laboratory analyses showed a similar pattern of findings: aHRs (95% CIs) 0.36 (0.17–0.76) and 0.16 (0.06–0.42) for the middle and highest tertiles, respectively.

### 2.4. Leptin and Mortality Risk

Similar to the L/A ratio analyses, incrementally higher leptin tertiles were associated with increasingly greater survival (reference: lowest leptin tertile) in both unadjusted (HRs [95% CIs] 0.45 [0.23–0.89] and 0.33 [0.15–0.70] for the middle and highest tertiles, respectively) and case-mix adjusted Cox regression analyses (aHRs [95% CIs] 0.30 [0.15–0.62] and 0.19 [0.08–0.43] for the middle and highest tertiles, respectively) (Figure 1B and Supplemental Table S5). Robust associations were observed in analyses incrementally adjusted for case-mix + laboratory covariates: aHRs (95% CIs) 0.39 (0.19–0.81) and 0.24 (0.10–0.57) for the middle and highest tertiles, respectively. In sensitivity analyses that adjusted for expanded case-mix + laboratory covariates, a similar pattern of findings was observed: aHRs (95% CIs) 0.44 (0.21–0.91) and 0.25 (0.10–0.60) for the middle and highest tertiles, respectively. These findings were robust in expanded case-mix + laboratory + BMI adjusted analyses: aHRs (95% CIs) 0.40 (0.19–0.88) and 0.20 (0.06–0.63) for the middle and highest leptin tertiles, respectively (Supplementary Table S6).

### 2.5. Adiponectin and Mortality Risk

In contrast to the L/A ratio and leptin analyses, we found that the highest adiponectin tertile was associated with higher death risk (reference: lowest adiponectin tertile) in both unadjusted (HRs [95% CIs] 3.03 [1.42–6.46]) and case-mix adjusted Cox regression analyses (aHRs [95% CIs] 2.83 [1.28–6.29]) (Figure 1C and Supplemental Table S5). Following incremental adjustment for case-mix + laboratory and expanded case-mix + laboratory covariates, point estimates for the highest adiponectin tertile suggested higher death risk, although estimates were no longer statistically significant: aHRs (95% CIs) 1.79 (0.76–4.18) and 2.00 (0.84–4.78) for case-mix + laboratory and expanded case-mix + laboratory adjusted analyses, respectively. Similar findings were observed in expanded case-mix + laboratory + BMI adjusted analyses: aHR (95% CI) 0.75 (0.27–2.03) and 1.79 (0.70–4.57) for the middle and highest adiponectin tertiles, respectively (Supplementary Table S6).

## 3. Discussion

In a well-defined multi-center prospective cohort of hemodialysis patients who underwent protocolized measurements of the uremic adipokines, leptin and adiponectin, we observed that there was a graded association between increasingly higher L/A ratios and greater survival benefit. These findings were robust across multiple secondary and sensitivity analyses that accounted for various socio-demographic, comorbidity, dialysis treatment, and laboratory parameter covariates. When adipokines were separately examined, we also found that incrementally higher leptin levels were associated with increasingly greater survival, whereas higher adiponectin levels were associated with higher death risk.

To date, there have been a sparse number of studies that have examined the relationship between leptin and survival in dialysis patients, which have shown mixed findings. In a prospective cohort of 71 hemodialysis patients from a single center in Germany, Scholze et al. reported that patients with lower serum leptin concentrations (i.e., below the median of observed levels) had worse survival compared with those with higher levels [19]. Notably, whereas lower serum leptin levels were observed in patients who suffered lethal cardiovascular events or those who were deceased at the end of the study (4.7 ± 9.4 µg/L and 5.2 ± 9.0 µg/L, respectively), the highest serum leptin concentrations were observed in event-free survivors (7.7 ± 7.8 µg/L). However, in a study of 205 patients with advanced stages 3–5 non-dialysis dependent CKD from Taiwan by Lu et al., a positive association between higher serum leptin levels and aortic stiffness measured by carotid-femoral pulse wave velocity was observed [20] Yet two other studies of maintenance hemodialysis patients from Taiwan and Israel conducted by Tsai et al. and Beberashvili et al., respectively, did not observe significant associations between serum leptin levels and cardiovascular outcomes nor mortality [21,22]. In contrast with leptin, there has been a comparatively larger body of literature examining adiponectin levels and clinical outcomes in dialysis patients, but these, too, have shown disparate findings. A prospective multi-center study of 501 US maintenance hemodialysis patients led by Rhee et al. showed that higher circulating adiponectin concentrations were associated with incrementally higher mortality risk [24]. Similarly, a subsequent study of 113 Japanese hemodialysis patients by Machiba et al. also found that higher serum adiponectin levels were linked with higher all-cause mortality risk [30]. Yet, in a study of 133 hemodialysis patients from Egypt, Abdallah et al. reported that plasma adiponectin levels demonstrated an inverse relationship with cardiovascular events and mortality [31]. Based on these collective data, the role of the adipokines, namely leptin and adiponectin, in the cardiovascular health and survival of CKD, and particularly hemodialysis, patients has remained unclear.

It has been suggested that the L/A ratio may be a better indicator of the risk of lethal cardiovascular disease, as it wholly considers the synergistic relationship of pro-atherogenic leptin and anti-atherogenic adiponectin within the context of one another. To date, there has only been one study examining L/A ratio and survival in dialysis patients. In a study by Park et al. of 131 non-diabetic ESKD patients on peritoneal dialysis from South Korea who underwent serum leptin and adiponectin measurements and were followed over a five-year period [29], higher L/A ratio levels were associated with higher all-cause mortality risk, whereas serum leptin and adiponectin levels examined separately were not significantly associated with mortality in multivariable Cox models adjusted for age and body mass index. However, extrapolation of these findings to the broader dialysis population is limited by its small cohort size, restriction to peritoneal dialysis patients from a single center, racial/ethnic homogeneity, and non-consideration of other key confounders of the adipokine—mortality relationship. Hence, to address these limitations, we conducted the first study of the synergistic role of leptin and adiponectin upon survival in a diverse, multi-center cohort of US maintenance HD patients, which showed robust associations between higher L/A ratio levels and lower mortality risk across four incremental levels of covariate adjustment. Given the disproportionate burden of cardiovascular mortality among in-center HD patients, there is a compelling need to identify novel, modifiable risk factors and therapeutic targets for cardiovascular disease and death in this population. Therefore, our findings may have important consequences with regard to the utilization of adipokines in both clinical practice and future research of ESKD patients.

It bears mentioning that the patterns of associations of high leptin and low adiponectin with improved survival in hemodialysis patients stand in contrast to observations in the non-CKD population, in whom leptin has been reported to be pro-atherogenic [10,11,12,13] and adiponectin as anti-atherogenic [32]. One potential factor influencing the paradoxical relationships observed in the hemodialysis cohort may relate to underlying nutritional status, including body fat stores and obesity status. For example, in a prospective cohort of 537 hemodialysis patients, Zoccali et al. observed that although higher adiponectin levels were associated with lower all-cause mortality among patients with the lowest waist circumference as a proxy of visceral body fat, higher adiponectin was associated with higher mortality among patients with the highest waist circumference [33]. When examining the body composition of hemodialysis patients, we previously found that higher body fat (i.e., subcutaneous adipose tissue, visceral adipose tissue, total body fat, and lean body mass) was associated with lower levels of adiponectin. Navaneethan et al. also reported that higher body fat was associated with higher levels of leptin and lower levels of adiponectin in their CKD patients [34]. These results point to the “obesity paradox” in dialysis patients [35,36,37], which unifies the counterintuitive associations between obesity and cardiovascular disease of many observational studies to propose that higher body fat may provide survival advantages in ESKD who are prone to malnutrition and protein–energy wasting. However, to better understand the paradoxical patterns of body fat, adipokines, and survival in CKD, further studies of the physiological mechanisms of adipokines and subsequent nutritional and cardioprotective impact in the dialysis population are needed. In normal metabolism, leptin binds with its ObRb receptors to inhibit synthesis of the appetite stimulant neuropeptide Y (NPY) [38], and adiponectin binds with its adipoR1 receptors to activate the AMPK pathway and its ATP formation processes, including fatty acid oxidation [39]. A subsequent analysis by Zoccali et al. found that higher serum NPY levels, possibly caused by lower leptin, predicted cardiovascular mortality in pre-dialysis patients [40]. Adiponectin and malnutrition were also correlated in a study of hemodialysis patients by Lee et al. [41]. Further studies, especially those on the fundamental biological level, will elucidate the sequence and causative factors among adipose tissue, adipokines, and nutritional physiology to define possible therapeutic targets.

Another noteworthy finding of our study was the relationship between adipokines and inflammatory markers observed in the logistic regression analyses. Higher serum leptin levels were associated with lower serum IL-6 concentrations, yet were associated with other markers of inflammation, namely lower serum albumin levels (i.e., negative acute phase reactant). Given that leptin has traditionally been considered a pro-inflammatory marker in the non-CKD/ESKD obese population (Supplementary Figure S3), these observations suggest that higher leptin levels may be associated with some (i.e., lower serum albumin), but not all (i.e., higher IL-6) inflammatory markers, and that the relationship between high leptin–low serum albumin may represent leptin’s relationship with specific inflammatory pathways. In the malnutrition-inflammation-cachexia hypothesis for the obesity paradox, patients with greater adipose tissue mass may be at lower risk of developing protein–energy wasting and its induction of an inflammatory state due to greater energy reserves. Given that higher leptin levels, as a by-product of increased fat mass, were associated with better survival in this dialysis cohort, it is possible that leptin’s favorable effects on nutritional status may overcome the ill effects of leptin-associated inflammation with respect to survival (i.e., higher leptin levels are a marker of obesity, which may paradoxically protect against the downstream effects of inflammation such as cardiovascular disease in dialysis patients) [42]. While serum albumin serves as both an inflammation marker and a nutritional marker [43], as the logistic regression analyses did not show that higher leptin was linked protein–energy wasting markers (i.e., nPCR nor serum creatinine as a proxy of muscle mass), we are less inclined to infer that the high leptin–low serum albumin association represents a link between leptin and poor nutritional status. Conversely, we found that higher serum adiponectin levels were associated with higher serum IL-6 levels in our dialysis cohort, which also stands in contrast to observations in the non-CKD/ESKD population (Supplementary Figure S3). Future studies are needed to elucidate the associations between adipokines, inflammation, and nutritional status for these adipokines to be utilized as biomarkers in CKD.

Similar to the general population, we also found that there were sex differences in leptin and adiponectin levels. For example, logistic regression analyses showed that female dialysis patients were more likely to have higher leptin levels, which is similar to observations in the non-CKD population, in which higher leptin concentrations in women have been attributed to greater fat mass and sex hormone differences (i.e., estrogen increases leptin production [44,45]). We also found that female dialysis patients were more likely to have higher adiponectin concentrations vs. their male counterparts; these observations are consistent with experimental models and human non-CKD studies in which testosterone reduces adiponectin levels [46,47]. When examining the baseline crude distribution of the L/A ratio, we also found that there was an increasingly high proportion of female patients across higher L/A ratio tertiles. Although the moderate sample size of this cohort limited the statistical power to perform sex-specific subgroup analyses, further research is warranted to explore potential sex-based differences in the associations between adipokines and survival in the dialysis population.

The strengths of this study include its prospective examination of a well-defined multi-center cohort of hemodialysis patients with case-mix characteristics similar to those of the broader US hemodialysis population; comprehensive availability of detailed patient-level data on socio-demographics, comorbid conditions, and dialysis treatment characteristics; and protocolized, uniform laboratory measurements of serum leptin and adiponectin in one centralized laboratory. However, several limitations bear mentioning. First, leptin and adiponectin serum concentrations were based on single measurements at the time of study entry, and thus changes in adipokine levels over time were not considered. Second, our adiponectin measurements did not differentiate between high and low molecular weight isoforms, which are associated with differential biological activity [48,49,50]. Third, we lacked information on cause-specific mortality (i.e., cardiovascular death) to gain greater insight into mechanistic pathways (i.e., atherogenic, cardio-metabolic [51]) by which adipokines impact mortality in hemodialysis patients. Fourth, the leptin and adiponectin measurements were variably measured following the two- and three-day interdialytic intervals; this may have resulted in residual confounding as these molecules may not be efficiently removed by conventional hemodialysis methods. Lastly, as with all observational studies, we cannot exclude the possibility of residual confounding. Future studies are needed to determine the causal relationships and underlying mechanisms of adipokines and outcomes in the dialysis population.

In summary, our study shows that higher serum L/A ratio levels are associated with greater survival in a diverse, multi-center prospective cohort of hemodialysis patients. Future studies are needed to confirm findings and define the underlying mechanisms by which high leptin and low adiponectin levels synergistically impact survival in hemodialysis patients in order to inform the utilization of adipokines as clinical biomarkers and future therapeutic targets.

## 4. Materials and Methods

### 4.1. Study Population

The study population was comprised of a cohort of maintenance hemodialysis patients enrolled in the NIH Malnutrition, Diet, and Racial Disparities in Chronic Kidney Disease (MADRAD) study (ClinicalTrials.gov study number: NCT01415570). The MADRAD study is a prospective cohort study examining differential associations between dietary factors and nutritional status with clinical outcomes across racial and ethnic hemodialysis subgroups. In this substudy of the MADRAD cohort, patients were recruited from 13 dialysis clinics in Southern California from October 2011 through February 2013. Patients in this substudy were included provided that they were ages 18–85 years, received in-center hemodialysis treatment for at least four consecutive weeks, had undergone protocolized serum leptin and adiponectin measurements, and signed a local institutional review board-approved consent form. Patients were excluded if they were actively receiving peritoneal dialysis, had a life expectancy of less than six months, or were unable to provide written consent without a proxy. The study was approved by the institutional review board committee of the University of California, Irvine.

### 4.2. Exposure Ascertainment

The exposure of interest was the ratio of serum leptin and adiponectin concentrations (i.e., L/A ratio). Serum leptin and adiponectin levels were measured from thawed serum samples that had been obtained pre-dialysis during weekday hemodialysis treatment sessions and subsequently frozen. Serum leptin and adiponectin levels (R&D Systems, Minneapolis, MN, USA) were measured at the University of California, Irvine, Institute of Clinical and Translational Science Bioassay Core.

### 4.3. Socio-Demographic, Comorbidity, and Laboratory Test Measures

Information on socio-demographics, comorbidities, medications, and hemodialysis treatment characteristics (e.g., vascular access type) was collected at study entry and every six months thereafter by the MADRAD research coordinators and study nephrologists (C.M.R., K.K.-Z.). Dialysis vintage was defined as the time period between the date of study entry and the date of hemodialysis initiation. Routine dialysis laboratory measurements were performed by the outpatient dialysis laboratories on a monthly or quarterly basis using automated methods. Protocolized serum IL-6 measurements were conducted using a human IL-6 ELISA high-sensitivity assay (Affymetrix). In this study, baseline values of routine laboratory tests were used.

### 4.4. Outcome Ascertainment

The primary outcome of interest was all-cause mortality. At-risk time began the day after serum leptin or adiponectin measurement, and patients were censored for kidney transplantation, transfer to non-affiliated dialysis clinic or peritoneal dialysis, or at the end of the substudy (21 January 2015). Each semester, information regarding mortality, censoring events, and associated dates from the preceding six months was collected from event forms completed by the MADRAD research coordinators and was reviewed by two MADRAD study nephrologists (C.M.R., K.K.-Z.).

### 4.5. Statistical Methods

Baseline characteristics between exposure groups were compared using chi-squared, analysis of variance (ANOVA), and Kruskal–Wallis tests as dictated by data type. We first examined the relationship of relevant clinical characteristics with high serum leptin and adiponectin levels (defined as the highest tertiles of observed values) separately using logistic regression.

We then estimated the association between serum L/A ratio, adiponectin, and leptin tertiles with all-cause mortality using Cox proportional hazard models with four incremental levels of covariate adjustment:

1. *Unadjusted model*: No adjustment for covariates;
2. *Case-mix adjusted model*: Adjusted for age, sex, race (Black vs. Non-Black race), ethnicity (Hispanic vs. Non-Hispanic ethnicity), diabetes status, dialysis vintage, and vascular access;
3. *Case-mix + laboratory adjusted model*: Adjusted for covariates in the case-mix model as well as serum albumin, interleukin-6 (IL-6), serum creatinine, and normalized protein catabolic rate (nPCR);
4. *Expanded case-mix + laboratory adjusted model*: Adjusted for covariates in the “case-mix + laboratory” model as well as calcium, phosphorus, parathyroid hormone (PTH), hemoglobin, and ferritin levels.

We a priori defined the “case-mix + laboratory” model as our primary model, which forced into the model core socio-demographic, comorbidity, and laboratory covariates. To explore the impact of other potential dialysis laboratory test confounders, we also conducted “expanded case-mix + laboratory” adjusted models as sensitivity analyses, given the high number of parameters relative to the number of death events. To determine the impact of body mass index (BMI) as a key confounder of the adipokine–mortality association, we also examined expanded “case-mix + laboratory + BMI” adjusted models as sensitivity analyses.

There were no missing data for age, sex, race/ethnicity, dialysis vintage, diabetes status, and IL-6 levels; remaining covariates had <1% missing values, except for vascular access (27%), serum albumin (14%), serum creatinine (16%), nPCR (11%), calcium (11%), phosphorus (11%), PTH (19%), hemoglobin (11%), ferritin (19%), and BMI (10%), which were handled using multiple imputation. The proportional hazards assumption was confirmed graphically and through Schoenfeld residual function testing. Analyses were carried out using statistical software Stata version 12.0 (StataCorp LP, College Station, TX, USA) and SAS version 9.4 (SAS Institute Inc., Cary, NC, USA).

## Figures and Tables

**Table 1:** Baseline characteristics of patients according to tertiles of leptin-to-adiponectin ratio.

		LEPTIN-TO-ADIPONECTIN RATIO CATEGORY			
	Overall	Tertile 1	Tertile 2	Tertile 3	*p*-Value *
No. of patients (%)	448 (100)	149 (33)	150 (33)	149 (33)	N/A
Leptin/adiponectin range (pg/mL)	<0.1–102.0	<0.1–<0.5	≥0.5–<3.2	≥3.20–102.0	N/A
Age (years) Mean ± SD	55.1 ± 14.3	52.8 ± 14.4	56.5 ± 14.9	56.1 ± 13.4	0.08
Female (%)	44	35	41	57	<0.001
Black race (%)	31	36	27	30	0.18
Hispanic ethnicity (%)	50	49	55	46	0.29
Diabetes (%)	55	48	55	60	0.11
AVF/AVG (%)	81	77	76	88	0.03
Vintage, months Median (IQR)	48.3 (27.6, 84.3)	53.0 (32.3, 89.9)	48.9 (23.9, 86.8)	42.3 (27.5, 78.2)	0.21
Laboratory Tests Median (IQR)					
Albumin (g/dL)	3.9 (3.7, 4.1)	4.0 (3.8, 4.2)	4.0 (3.7, 4.2)	3.9 (3.7, 4.0)	0.14
Creatinine (mg/dL)	10.0 (8.2, 12.3)	10.6 (8.4, 13.3)	9.3 (7.8, 11.2)	10.0 (8.3, 11.9)	0.02
Corrected calcium (mg/dL)	9.1 (8.7, 9.5)	9.0 (8.5, 9.5)	9.1 (8.7, 9.4)	9.1 (8.8, 9.5)	0.38
Phosphorus (mg/dL)	5.2 (4.3, 6.3)	5.3 (4.4, 6.8)	5.1 (4.1, 6.4)	5.0 (4.3, 6.0)	0.06
Intact PTH (pg/mL)	385 (263, 579)	435 (279, 663)	362 (256, 510)	392 (256, 580)	0.12
Hemoglobin (g/dL)	10.7 (10.1, 11.4)	10.7 (10.1, 11.2)	10.7 (10.1, 11.5)	10.8 (10.2, 11.4)	0.67
Ferritin (ng/mL)	638 (415, 865)	699 (404, 873)	599 (413, 843)	622 (429, 845)	0.92
nPCR (g/kg/day)	1.0 (0.9, 1.2)	1.0 (0.9, 1.2)	1.0 (0.8, 1.2)	1.0 (0.9, 1.2)	0.68
Adiponectin (mcg/mL)	15.1 (9.1, 24.2)	24.5 (18.0, 32.7)	14.9 (10.4, 22.6)	8.3 (5.1, 12.8)	<0.001
Leptin (mcg/mL)	16.5 (5.7, 54.6)	3.4 (0.8, 6.4)	16.7 (11.0, 23.1)	112.0 (49.6, 168.4)	<0.001
IL-6	2.3 (1.3, 4.2)	2.8 (1.4, 6.3)	2.1 (1.0, 3.8)	2.2 (1.4, 4.0)	0.004

Note: Categorical variables are given as number (percentage); continuous variables, as mean ± standard deviation or median (IQR). * *p*-value calculated by chi-square, ANOVA, or Kruskal–Wallis tests. Abbreviations: AVF, arteriovenous fistula; AVG, arteriovenous graft; IL-6, interleukin-6; IQR, interquartile range; N/A, not applicable; nPCR, normalized protein catabolic rate; PTH, parathyroid hormone; SD, standard deviation.

**Table 2:** Clinical characteristics associated with the highest leptin tertile (vs. lowest two tertiles) using logistic regression.

	Unadjusted		Case-Mix Adjusted *		Case-Mix + Laboratory **	
	OR (95% CI)	*p*-Value	OR (95% CI)	*p*-Value	OR (95% CI)	*p*-Value
Age (∆10 years)	1.05 (0.91, 1.20)	0.51	0.96 (0.82, 1.13)	0.63	0.96 (0.80, 1.14)	0.62
Vintage (∆1 year)	1.01 (0.96, 1.06)	0.65	1.00 (0.95, 1.06)	0.91	1.01 (0.95, 1.07)	0.75
Female	3.66 (2.42, 5.53)	<0.001	3.79 (2.48, 5.80)	<0.001	3.58 (2.23, 5.77)	<0.001
Black race	0.96 (0.63, 1.47)	0.85	0.74 (0.41, 1.31)	0.30	0.65 (0.35, 1.22)	0.18
Hispanic	0.81 (0.55, 1.20)	0.30	0.66 (0.39, 1.13)	0.13	0.61 (0.35, 1.07)	0.08
Diabetes	1.27 (0.85, 1.89)	0.24	1.55 (0.96, 2.50)	0.07	1.69 (1.04, 2.76)	0.04
AVF/AVG (vs. catheter)	2.01 (1.07, 3.78)	0.03	2.23 (1.14, 4.37)	0.02	2.20 (1.10, 4.39)	0.03
Serum albumin (∆0.5 g/dL)	0.72 (0.54, 0.96)	0.03	0.74 (0.53, 1.02)	0.07	0.59 (0.41, 0.84)	0.004
Creatinine (mg/dL)	0.96 (0.90, 1.03)	0.29	1.05 (0.95, 1.15)	0.35	1.04 (0.94, 1.15)	0.45
Calcium (mg/dL)	1.10 (0.79, 1.52)	0.57	0.97 (0.68, 1.39)	0.86	1.01 (0.70, 1.46)	0.98
Phosphorus (mg/dL)	0.90 (0.78, 1.03)	0.11	0.92 (0.80, 1.07)	0.28	0.91 (0.77, 1.07)	0.26
PTH (pg/mL)	1.00 (1.00, 1.00)	0.61	1.00 (1.00, 1.00)	0.69	1.00 (1.00, 1.00)	0.58
Hemoglobin (g/dL)	1.04 (0.86, 1.27)	0.67	1.16 (0.94, 1.44)	0.17	1.15 (0.92, 1.44)	0.22
Ferritin (ng/mL)	1.00 (1.00, 1.00)	0.73	1.00 (1.00, 1.00)	0.23	1.00 (1.00, 1.00)	0.45
nPCR (g/kg/day)	1.32 (0.64, 2.74)	0.45	1.24 (0.55, 2.79)	0.60	1.15 (0.48, 2.71)	0.76
Adiponectin (∆10)	0.46 (0.36, 0.59)	<0.001	0.30 (0.22, 0.41)	<0.001	0.30 (0.22, 0.41)	<0.001
IL-6 (∆5)	0.57 (0.40, 0.82)	0.002	0.57 (0.39, 0.85)	0.006	0.50 (0.33, 0.77)	0.002

Abbreviations: AVF, arteriovenous fistula; AVG, arteriovenous graft; CI, confidence interval; IL-6, interleukin-6; nPCR, normalized protein catabolic rate; PTH, parathyroid hormone. * Case-mix analyses adjusted for age, sex, race, ethnicity, diabetes, dialysis vintage, and access. ** Case-mix + laboratory analyses adjusted for age, sex, race, ethnicity, diabetes, dialysis vintage, access, albumin, IL-6, creatinine, and nPCR.

**Table 3:** Clinical characteristics associated with the highest adiponectin tertile (vs. lowest two tertiles) using logistic regression.

	Unadjusted		Case-Mix Adjusted *		Case-Mix + Laboratory **	
	OR (95% CI)	*p*-Value	OR (95% CI)	*p*-Value	OR (95% CI)	*p*-Value
Age (∆10 years)	1.06 (0.92, 1.22)	0.40	1.06 (0.91, 1.24)	0.46	1.02 (0.86, 1.21)	0.80
Vintage (∆1 year)	1.10 (1.05, 1.16)	<0.001	1.10 (1.04, 1.16)	<0.001	1.10 (1.04, 1.16)	<0.001
Female	1.93 (1.30, 2.87)	0.001	1.89 (1.26, 2.84)	0.002	1.94 (1.24, 3.05)	0.004
Black race	1.46 (0.96, 2.21)	0.08	1.58 (0.90, 2.78)	0.11	1.83 (1.01, 3.34)	0.05
Hispanic	0.95 (0.64, 1.41)	0.81	1.36 (0.79, 2.32)	0.27	1.40 (0.81, 2.43)	0.23
Diabetes	0.92 (0.62, 1.36)	0.66	1.03 (0.66, 1.63)	0.89	0.94 (0.59, 1.49)	0.78
AVF/AVG	0.98 (0.54, 1.77)	0.95	0.82 (0.44, 1.54)	0.54	0.85 (0.45, 1.62)	0.62
Serum albumin (∆0.5 g/dL)	0.78 (0.58, 1.03)	0.08	0.81 (0.60, 1.10)	0.18	0.92 (0.66, 1.28)	0.63
Creatinine (mg/dL)	0.94 (0.87, 1.01)	0.07	0.96 (0.88, 1.04)	0.29	0.96 (0.88, 1.05)	0.35
Calcium (mg/dL)	1.07 (0.78, 1.47)	0.69	0.98 (0.70, 1.36)	0.88	0.97 (0.69, 1.35)	0.84
Phosphorus (mg/dL)	1.01 (0.89, 1.15)	0.91	1.05 (0.91, 1.20)	0.51	1.05 (0.91, 1.22)	0.50
PTH (pg/mL)	1.00 (1.00, 1.00)	0.73	1.00 (1.00, 1.00)	0.88	1.00 (1.00, 1.00)	0.89
Hemoglobin (g/dL)	0.86 (0.71, 1.04)	0.13	0.88 (0.72, 1.08)	0.24	0.92 (0.75, 1.14)	0.46
Ferritin (ng/mL)	1.00 (1.00, 1.00)	0.13	1.00 (1.00, 1.00)	0.31	1.00 (1.00, 1.00)	0.40
nPCR (g/kg/day)	1.33 (0.65, 2.73)	0.44	1.61 (0.75, 3.43)	0.22	1.96 (0.89, 4.34)	0.10
Leptin (∆25)	0.74 (0.66, 0.84)	<0.001	0.66 (0.57, 0.76)	<0.001	0.66 (0.57, 0.76)	<0.001
IL-6 (∆5)	1.40 (1.07, 1.83)	0.01	1.57 (1.18, 2.09)	0.002	1.55 (1.15, 2.08)	0.004

Abbreviations: AVF, arteriovenous fistula; AVG, arteriovenous graft; CI, confidence interval; IL-6, interleukin-6; nPCR, normalized protein catabolic rate; PTH, parathyroid hormone. * Case-mix analyses adjusted for age, sex, race, ethnicity, diabetes, dialysis vintage, and access. ** Case-mix + laboratory analyses adjusted for age, sex, race, ethnicity, diabetes, dialysis vintage, access, albumin, IL-6, creatinine, and nPCR.

## Data Availability

Due to the nature of the research and restrictions (i.e., data containing information that could compromise the privacy of research participants), supporting data are not available.

## Supplementary Materials

The following supporting information can be downloaded at: https://www.mdpi.com/article/10.3390/toxins17110525/s1, Figure S1. Study cohort creation algorithm. Figure S2. Scatterplot of leptin and adiponectin levels. An overlying regression line of the relationship between leptin and adiponectin is shown in red (adiponectin = 19.90385 − 0.0443667 × leptin). Figure S3. Hypothesized relationship between adipokines and IL-6 in the general population vs. dialysis population. Table S1. Baseline characteristics of patients according to tertiles of leptin. Table S2. Baseline characteristics of patients according to tertiles of adiponectin. Table S3. Spearman correlation coefficients (R) of clinical characteristics and serum leptin levels. Table S4. Spearman correlation coefficients (R) of clinical characteristics and serum adiponectin levels. Table S5. Associations of leptin-to-adiponectin (L/A) ratio, leptin, and adiponectin with mortality using Cox regression. Table S6. Sensitivity analyses of the associations of leptin and adiponectin with mortality using Cox regression accounting for body mass index.
