# Supplementary material for: Leptin and Adiponectin as Uremic Adipokines: Associations with Survival in a Prospective Hemodialysis Cohort
Source: Toxins (Basel). 2025 Oct 25;17(11):525. doi: 10.3390/toxins17110525 (PMC12656480; doi:10.3390/toxins17110525)

Table S1. Baseline characteristics of patients according to tertiles of leptin.

		LEPTIN CATEGORY			
	**Overall**	**Tertile 1**	**Tertile 2**	**Tertile 3**	**p-value***
No. of patients (%)	448 (100)	149 (33)	150 (33.48)	149 (33.26)	N/A
Leptin range (pg/ml)	<0.1-401.50	<0.1-<7.9	≥7.9-<31.8	≥31.80-401.5	N/A
Age (years)<br>Mean ± SD	55.1±14.3	53.1±14.5	56.6±13.7	55.7±14.5	0.12
Female (%)	44	28	39	65	<0.001
Black race (%)	31	36	26	30	0.16
Hispanic ethnicity (%)	50	48	55	46	0.32
Diabetes (%)	54	49	56	58	0.24
AVF/AVG (%)	81	77	77	87	0.09
Vintage, months<br>Median (IQR)	48.3 (27.6, 84.3)	51.1 (27.5, 89.9)	48.2 (23.0, 81.7)	46.4 (29.6, 80.6)	0.59
**Laboratory Tests<br>Median (IQR)**					
Albumin (g/dl)	3.9 (3.7, 4.1)	3.9 (3.8, 4.2)	4.0 (3.7, 4.2)	3.9 (3.7, 4.0)	0.03
Creatinine (mg/dl)	10.0 (8.2, 12.3)	10.3 (8.5, 13.2)	9.4 (7.6, 11.7)	9.8 (8.2, 11.7)	0.07
Corrected Calcium (mg/dl)	9.1 (8.7, 9.5)	9.0 (8.6, 9.5)	9.1 (8.8, 9.4)	9.1 (8.7, 9.5)	0.60
Phosphorus (mg/dl)	5.2 (4.3, 6.3)	5.2 (4.4, 6.5)	5.1 (4.3, 6.4)	5.2 (4.3, 6.0)	0.40
Intact PTH (pg/ml)	385 (263, 579)	431 (287, 728)	354 (253, 520)	385 (256, 581)	0.09
Hemoglobin (g/dl)	10.7 (10.1, 11.4)	10.7 (10.1, 11.3)	10.8 (10.1, 11.4)	10.8 (10.1, 11.4)	0.74
Ferritin (ng/ml)	638 (415, 865)	686 (426, 862)	629 (411, 870)	611 (417, 844)	0.92
nPCR (g/kg/day)	1.0 (0.9, 1.2)	1.0 (0.9, 1.2)	1.0 (0.8, 1.2)	1.0 (0.9, 1.2)	0.58
Adiponectin (mcg/mL)	15.1 (9.1, 24.2)	21.3 (14.1, 31.0)	14.9 (9.7, 23.9)	10.3 (7.1, 16.0)	<0.001
Leptin (mcg/mL)	2.3 (1.3, 4.2)	2.8 (1.4, 6.3)	2.2 (1.1, 4.2)	2.2 (1.4, 3.7)	0.01
IL-6	3.9 (3.7, 4.1)	3.9 (3.8, 4.2)	4.0 (3.7, 4.2)	3.9 (3.7, 4.0)	0.03

Note: Categorical variables are given as number (percentage); continuous variables, as mean ± standard deviation or median (IQR).
*P-value calculated by analysis of variance, chi-square, or Kruskal-Wallis tests.
Abbreviations: AVF, arteriovenous fistula; AVG, arteriovenous graft; IL-6, interleukin-6; IQR, interquartile range; N/A, not applicable; nPCR, normalized protein catabolic rate; PTH, parathyroid hormone; SD, standard deviation.

Table S2. Baseline characteristics of patients according to tertiles of adiponectin.

		ADIPONECTIN CATEGORY			
	**Overall**	**Tertile 1**	**Tertile 2**	**Tertile 3**	**p-value***
No. of patients (%)	448 (100)	149 (33)	150 (33)	149 (33)	NA
Adiponectin range (pg/ml)	1.6-79.5	1.64-<11.0	≥11.0-<20.8	≥20.8-79.5	NA
Age (years)<br>Mean ± SD	55.1±14.3	53.1±11.9	56.3±15.3	55.9±15.2	0.04
Female (%)	44	47 (31.54)	69 (46.00)	82 (55.03)	<0.001
Black race (%)	31	47 (31.54)	37 (24.67)	54 (36.24)	0.09
Hispanic ethnicity (%)	50	66 (44.30)	84 (56.00)	73 (48.99)	0.13
Diabetes (%)	54	78 (52.35)	87 (58.00)	79 (53.02)	0.56
AVF/AVG (%)	81	98 (85.96)	82 (75.23)	82 (80.39)	0.13
Vintage, months<br>Median (IQR)	48.3 (27.6, 84.3)	39.0 (22.1, 72.9)	45.1 (22.5, 79.0)	59.4 (38.6, 95.4)	<0.001
**Laboratory Tests<br>Median (IQR)**					
Albumin (g/dl)	3.9 (3.7, 4.1)	4.0 (3.8, 4.2)	3.9 (3.8, 4.2)	3.9 (3.7, 4.1)	0.13
Creatinine (mg/dl)	10.0 (8.2, 12.3)	10.9 (8.5, 12.6)	9.7 (8.2, 12.2)	9.5 (7.9, 11.5)	0.04
Corrected Calcium (mg/dl)	9.1 (8.7, 9.5)	9.1 (8.8, 9.5)	9.1 (8.6, 9.4)	9.1 (8.6, 9.6)	0.49
Phosphorus (mg/dl)	5.2 (4.3, 6.3)	5.1 (4.2, 6.1)	5.2 (4.4, 6.4)	5.1 (4.2, 6.5)	0.61
Intact PTH (pg/ml)	385 (263, 579)	388 (265, 540)	377 (280, 594)	409 (243, 599)	0.97
Hemoglobin (g/dl)	10.7 (10.1, 11.4)	10.8 (10.2, 11.5)	10.8 (10.1, 11.3)	10.7 (10.1, 11.3)	0.27
Ferritin (ng/ml)	638 (415, 865)	598 (408, 791)	690 (391, 872)	674 (445, 892)	0.15
nPCR (g/kg/day)	1.0 (0.9, 1.2)	1.0 (0.9, 1.2)	1.0 (0.9, 1.2)	1.0 (0.9, 1.2)	0.87
Adiponectin (mcg/mL)	16.5 (5.7, 54.6)	34.7 (12.4, 142.3)	16.2 (5.9, 51.3)	7.5 (2.7, 18.4)	<0.001
Leptin (mcg/mL)	2.3 (1.3, 4.2)	2.1 (1.1, 3.8)	2.2 (1.3, 4.2)	2.6 (1.4, 5.2)	0.06
IL-6	3.9 (3.7, 4.1)	4.0 (3.8, 4.2)	3.9 (3.8, 4.2)	3.9 (3.7, 4.1)	0.13

Note: Categorical variables are given as number (percentage); continuous variables, as mean ± standard deviation or median (IQR).
*P-value calculated by analysis of variance, chi-square, or Kruskal-Wallis tests.
Abbreviations: AVF, arteriovenous fistula; AVG, arteriovenous graft; IL-6, interleukin-6; IQR, interquartile range; N/A, not applicable; nPCR, normalized protein catabolic rate; PTH, parathyroid hormone; SD, standard deviation.

Table S3. Spearman correlation coefficients (R) of clinical characteristics and serum leptin levels.

	Unadjusted		Case-Mix*		Case-Mix + Laboratory**	
**Variable**	**R**	**p-value**	**R**	**p-value**	**R**	**p-value**
Age	**0.10**	**0.03**	0.05	0.37	0.07	0.30
Vintage	-0.01	0.89	0.003	0.96	-0.009	0.89
**Laboratory Tests**						
Serum albumin (g/dl)	**-0.15**	**0.003**	-0.09	0.17	-0.11	0.07
Creatinine (mg/dl)	**-0.12**	**0.02**	0.02	0.80	0.008	0.90
Calcium (mg/dl)	0.07	0.19	0.05	0.38	0.07	0.29
Phosphorus (mg/dl)	-0.07	0.14	-0.03	0.57	-0.04	0.53
PTH (pg/ml)	-0.04	0.44	-0.01	0.83	-0.05	0.45
Hemoglobin (g/dl)	0.02	0.67	0.05	0.43	0.06	0.34
Ferritin (ng/ml)	-0.02	0.66	-0.10	0.11	-0.10	0.13
nPCR (g/kg/day)	0.06	0.20	0.06	0.35	0.07	0.25
Adiponectin	**-0.39**	**<0.001**	**-0.54**	**<0.001**	**-0.54**	**<0.001**
IL-6	**-0.13**	**0.007**	**-0.07**	**0.21**	**-0.14**	**0.03**

*Case-mix analyses adjusted for age, sex, race, ethnicity, diabetes, dialysis vintage, and access.

**Case-mix+laboratory analyses adjusted for age, sex, race, ethnicity, diabetes, dialysis vintage, access, albumin, IL-6, creatinine, and nPCR.

Abbreviations: BMI, body mass index; IL-6, interleukin-6; NIR, near-infrared; nPCR, normalized protein catabolic rate; PTH, parathyroid hormone.

Table S4. Spearman correlation coefficients (R) of clinical characteristics and serum adiponectin levels.

	**Unadjusted**		**Case-Mix***		**Case-Mix + Laboratory****	
**Variable**	**R**	**p-value**	**R**	**p-value**	**R**	**p-value**
Age	0.08	0.10	0.03	0.59	0.02	0.79
Vintage	**0.21**	**<0.001**	**0.20**	**<0.001**	**0.20**	**0.002**
**Laboratory Tests**						
Serum albumin (g/dl)	-0.09	0.09	-0.04	0.56	0.02	0.79
Creatinine (mg/dl)	**-0.13**	**0.01**	-0.10	0.11	-0.09	0.18
Calcium (mg/dl)	-0.03	0.58	-0.05	0.40	-0.07	0.31
Phosphorus (mg/dl)	0.04	0.45	0.06	0.31	0.08	0.21
PTH (pg/ml)	0.03	0.57	-0.06	0.40	-0.01	0.86
Hemoglobin (g/dl)	-0.08	0.12	-0.08	0.18	-0.07	0.26
Ferritin (ng/ml)	**0.13**	**0.02**	0.12	0.07	0.13	0.06
nPCR (g/kg/day)	-0.02	0.62	-0.02	0.75	-0.02	0.79
Leptin	**-0.39**	**<0.001**	**-0.54**	**<0.001**	**-0.54**	**<0.001**
IL-6	**0.11**	**0.03**	0.10	0.07	**0.13**	**0.04**

*Case-mix analyses adjusted for age, sex, race, ethnicity, diabetes, dialysis vintage, and access.
**Case-mix+laboratory analyses adjusted for age, sex, race, ethnicity, diabetes, dialysis vintage, access, albumin, IL-6, creatinine, and nPCR.
Abbreviations: BMI, body mass index; IL-6, interleukin-6; NIR, near-infrared; nPCR, normalized protein catabolic rate; PTH, parathyroid hormone.

Table S5. Associations of leptin-to-adiponectin (L/A) ratio, leptin, and adiponectin with mortality using Cox regression.

LEPTIN-TO-ADIPONECTIN RATIO ANALYSES								
	**Unadjusted**		**Case-Mix***		**Case-Mix+Laboratory****		**Expanded Case-Mix+Lab*****	
**L/A Ratio**	**HR (95% CI)**	**p-value**	**HR (95% CI)**	**p-value**	**HR (95% CI)**	**p-value**	**HR (95% CI)**	**p-value**
Tertile 1	Reference	N/A	Reference	N/A	Reference	N/A	Reference	N/A
Tertile 2	0.46 (0.24-0.89)	0.02	0.28 (0.14-0.58)	<0.001	0.37 (0.18-0.76)	0.007	0.36 (0.17-0.76)	0.007
Tertile 3	0.24 (0.10-0.55)	<0.001	0.14 (0.06-0.35)	<0.001	0.18 (0.07-0.46)	<0.001	0.16 (0.06-0.42)	<0.001
**LEPTIN ANALYSES**								
	**Unadjusted**		**Case-Mix***		**Case-Mix+Laboratory****		**Expanded Case-Mix+Lab*****	
**Leptin**	**HR (95% CI)**	**p-value**	**HR (95% CI)**	**p-value**	**HR (95% CI)**	**p-value**	**HR (95% CI)**	**p-value**
Tertile 1	Reference	N/A	Reference	N/A	Reference	N/A	Reference	N/A
Tertile 2	0.45 (0.23-0.89)	0.02	0.30 (0.15-0.62)	0.001	0.39 (0.19-0.81)	0.01	0.44 (0.21-0.91)	0.03
Tertile 3	0.33 (0.15-0.70)	0.003	0.19 (0.08-0.43)	<0.001	0.24 (0.10-0.57)	0.001	0.25 (0.10-0.60)	0.002
**ADIPONECTIN ANALYSES**								
	**Unadjusted**		**Case-Mix***		**Case-Mix+Laboratory****		**Expanded Case-Mix+Lab*****	
**Adiponectin**	**HR (95% CI)**	**p-value**	**HR (95% CI)**	**p-value**	**HR (95% CI)**	**p-value**	**HR (95% CI)**	**p-value**
Tertile 1	Reference	N/A	Reference	N/A	Reference	N/A	Reference	N/A
Tertile 2	1.33 (0.56-3.16)	0.52	1.09 (0.45-2.66)	0.84	0.72 (0.28-1.87)	0.50	0.80 (0.30-2.14)	0.66
Tertile 3	3.03 (1.42-6.46)	0.004	2.83 (1.28-6.29)	0.01	1.79 (0.76-4.18)	0.18	2.00 (0.84-4.78)	0.12

*Case-mix analyses adjusted for age, sex, race, ethnicity, diabetes, dialysis vintage, and access.

**Case-mix+laboratory analyses adjusted for age, sex, race, ethnicity, diabetes, dialysis vintage, access, albumin, IL-6, creatinine, and nPCR.

***Expanded case-mix+laboratory analyses adjusted for age, sex, race, ethnicity, diabetes, dialysis vintage, access, albumin, IL-6, creatinine, nPCR, calcium, phosphorus, PTH, hemoglobin, and ferritin.

Abbreviations: CI, confidence interval; HR, hazard ratio; IL-6, interleukin-6; IQR, interquartile range; nPCR, normalized protein catabolic rate; PTH, parathyroid hormone.

Table S6. Sensitivity analyses of the associations of leptin and adiponectin with mortality using Cox regression accounting for body mass index.

LEPTIN ANALYSES		
	Expanded case-mix+laboratory+BMI analyses	
**Leptin**	**HR (95% CI)**	**p-value**
Tertile 1	Reference	N/A
Tertile 2	0.40 (0.19-0.88)	0.02
Tertile 3	0.20 (0.06-0.63)	0.007
**ADIPONECTIN ANALYSES**		
	**Expanded case-mix+laboratory+BMI analyses**	
**Adiponectin**	**HR (95% CI)**	**p-value**
Tertile 1	Reference	N/A
Tertile 2	0.75 (0.27-2.03)	0.57
Tertile 3	1.79 (0.70-4.57)	0.22

*Expanded case-mix+laboratory+BMI analyses adjusted for age, sex, race, ethnicity, diabetes, dialysis vintage, access, albumin, IL-6, creatinine, nPCR, calcium, phosphorus, PTH, hemoglobin, ferritin, and BMI.

Abbreviations: CI, confidence interval; HR, hazard ratio; IL-6, interleukin-6; IQR, interquartile range; nPCR, normalized protein catabolic rate; PTH, parathyroid hormone.

Figure S1. Study cohort creation algorithm.

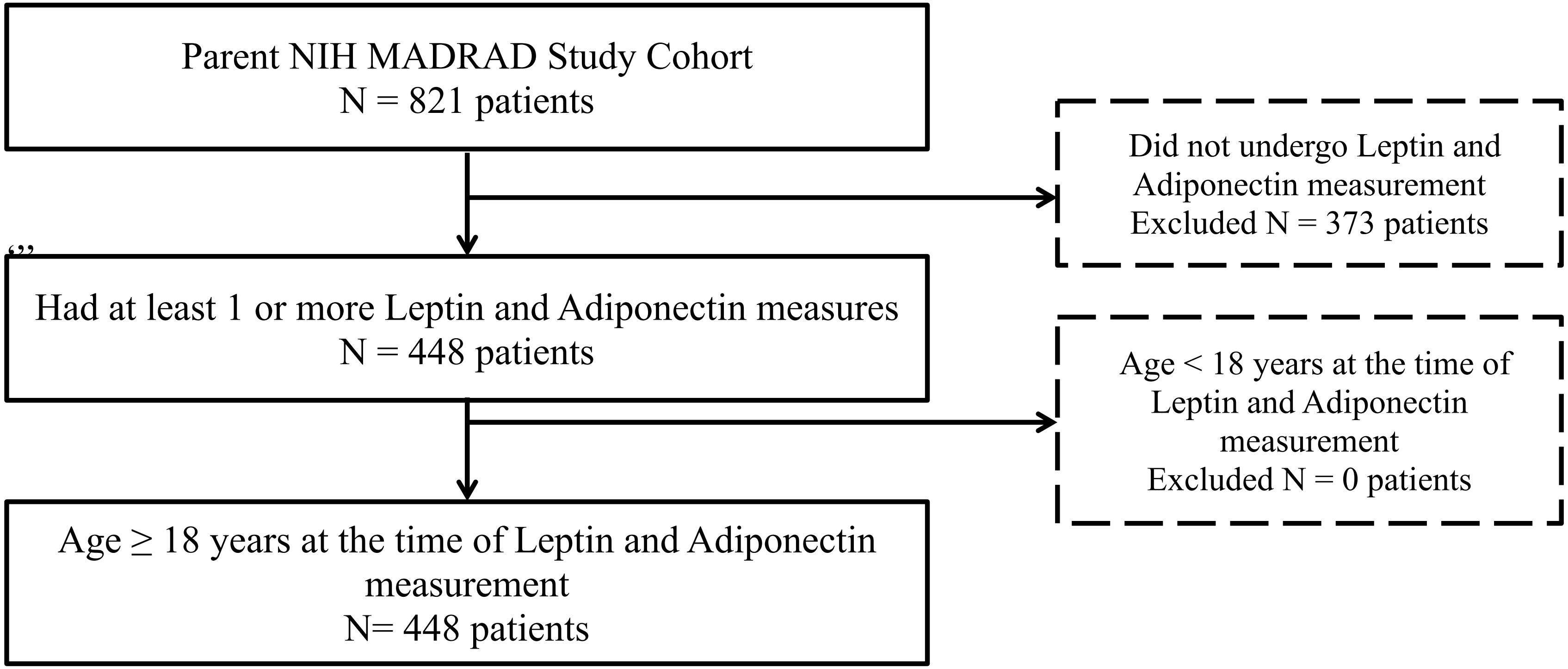

Figure S2. Scatterplot of leptin and adiponectin levels. *An overlying regression line of the relationship between leptin and adiponectin is shown in blue (adiponectin = 19.90385 -.0443667*leptin).*

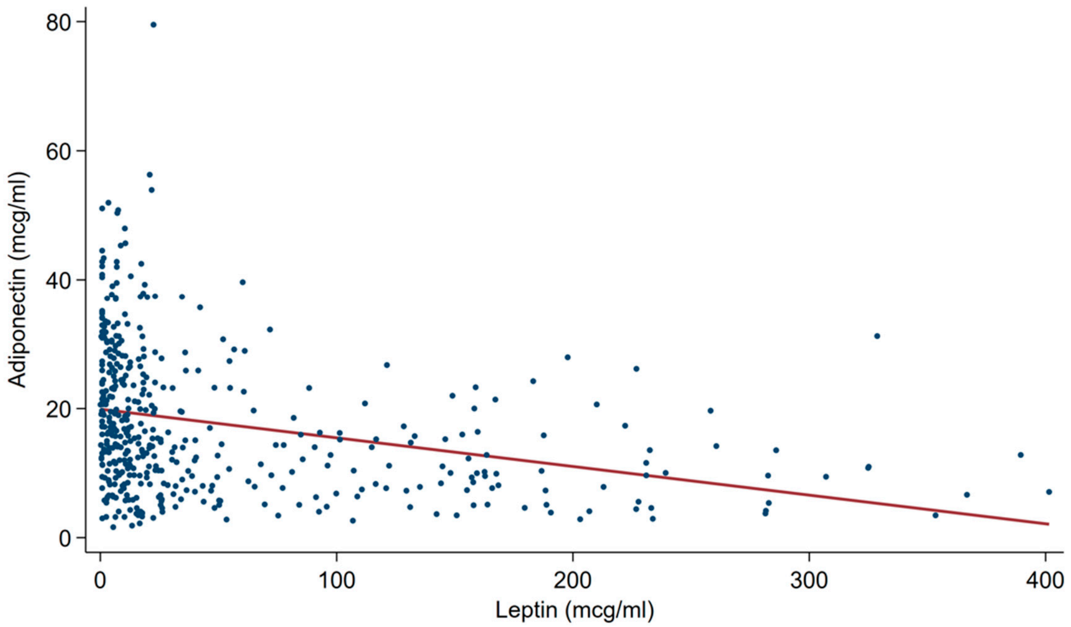

Figure S3. Hypothesized relationship between adipokines and IL-6 in the general population vs. dialysis population.

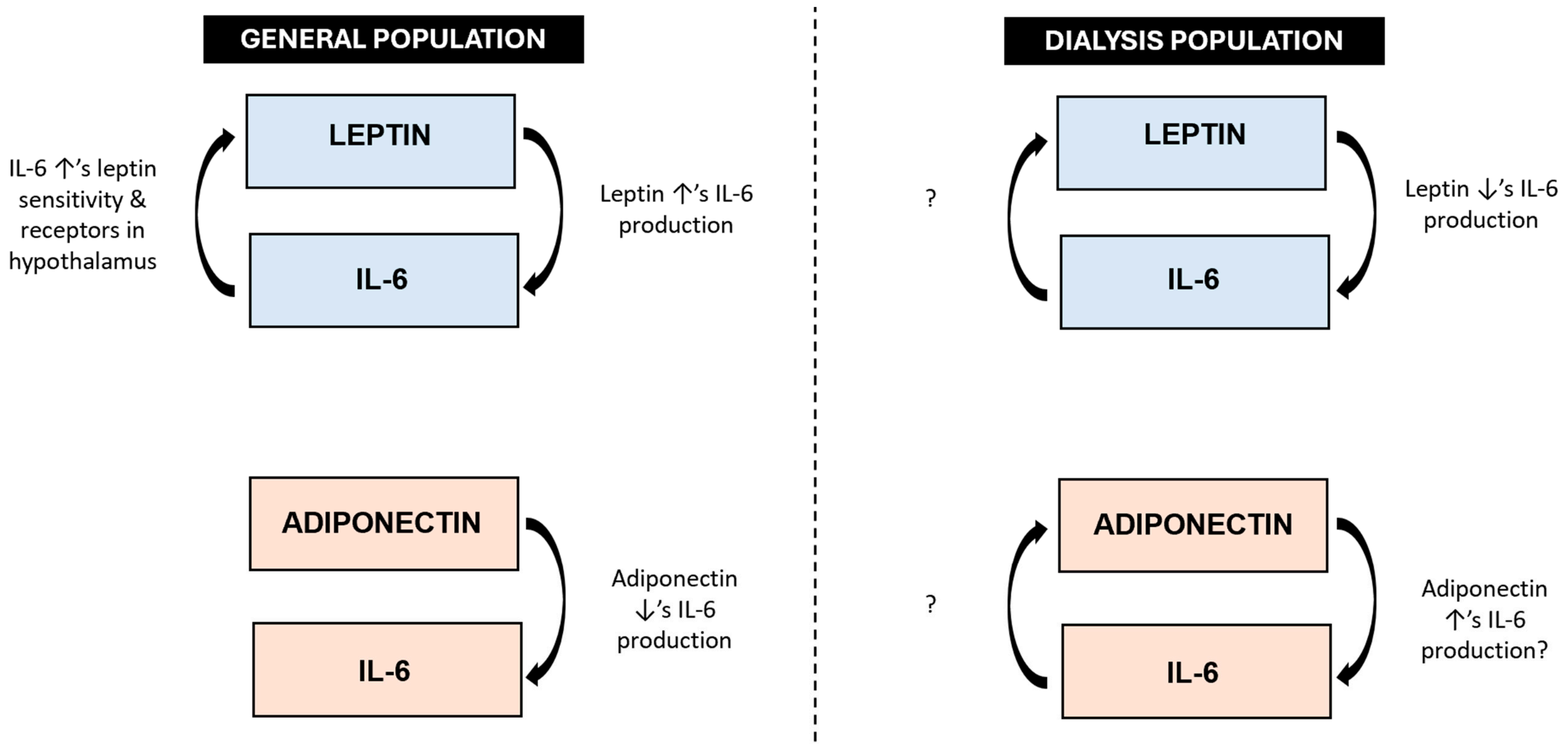

Supplement: Supplementary file 1 [file toxins-17-00525-s001.zip › toxins-3758527-supplementary.pdf]
